# Product Lifecycle Information Flow in E-waste Handling: a Means to Increase Circularity?

**DOI:** 10.1007/s43615-023-00258-1

**Published:** 2023-02-22

**Authors:** Terje Andersen, Lise Lillebrygfjeld Halse

**Affiliations:** Molde University College – Specialized University in Logistics, P.O. Box 2110, NO-6402 Molde, Norway

**Keywords:** e-waste, Waste Electrical and Electronic Equipment (WEEE), Product lifecycle information flow, Manufacturer, Circular supply chain management (CSCM)

## Abstract

Electronic waste (e-waste) is a growing waste stream. In Europe, e-waste is regulated by the Waste Electrical and Electronic Equipment (WEEE) Directive. Each manufacturer or importer is responsible for the end-of-life (EoL) treatment of the equipment it handles, although this task is usually outsourced to producer responsibility organizations (PROs) that collect and treat the e-waste. The WEEE regime has been criticized for focusing on waste handling according to the traditional linear economy, while, in a circular economy, the goal is to eliminate waste. Information sharing helps improve circularity, and digital technology is seen as enabling information transparency and visibility in the supply chain. However, there is need for empirical studies demonstrating the use of information in supply chains to improve circularity. We conducted a case study of a manufacturer, including its subsidiaries and PROs in eight European countries, in which we investigated the product lifecycle information flow related to e-waste. Our findings indicate that product lifecycle information is available, but that it is provided for purposes other than e-waste handling. Actors are willing to share this information, but it is not regarded as useful for EoL treatment since the actors involved in EoL handling believe that using this information could lead to delays and poorer performance in e-waste handling. Our findings contradict the optimistic view of digital technology as improving circularity in circular supply chain management. The findings further give reason to question the implementation of digital technology to improve the product lifecycle information flow as long as the involved actors do not request this information.

## Introduction

Electronic waste (e-waste) is among the world’s fastest-growing waste streams [1, 2]. This waste contains precious and hazardous metals, including copper, gold, silver, palladium, cobalt, phosphorous, and platinum. The extraction of these materials occupies vast areas, uses other precious resources, such as water and energy, and is responsible for significant CO_2_ emissions. The amount of e-waste has been increasing since the use of electrical and electronic equipment (EEE) with short lifetimes has become more prevalent [3]. This material has value that can be extracted via landfill mining [4, 5]. The annual value of landfill e-waste is estimated to be approximately USD 57 billion [2]. In Europe, e-waste is regulated by the Waste Electric and Electronic Equipment (WEEE) Directive [6]. The Directive is based on the extended producer responsibility principle [7], which means that each producer must take responsibility for the end-of-life (EoL) phase of the products it manufactures. However, the Directive allows producers to outsource e-waste handling to producer responsibility organizations (PROs) [6]. Such outsourcing has become the de facto standard for discharging the manufacturer’s responsibility for EoL e-waste treatment [8, 9]. Although e-waste treatment in Europe is trending toward recovery and recycling, the focus is still on waste handling [2]. Moreover, only 17% of the globally generated e-waste is properly collected and recycled [2]. To meet this challenge, information flow and digitalization have been regarded as important enablers to improve the circularity in EEE supply chains [10, 11]. This is the main issue addressed in this study.

E-waste handling has attracted considerable attention in different streams of the literature as environmental science, environmental engineering, green sustainable science and technology, chemical engineering and multidisciplinary chemistry [12]. An emerging literature stream applies the circular economy (CE) lens to the EEE supply chain, with EoL concepts being replaced with CE strategies such as reduce, reuse, remanufacture, and recycle (4R) [10, 13]. In the CE literature, several studies have advocated more circularity in e-waste treatment in both Europe and elsewhere [13–16]. The renewed attention to the CE has led to a call for more research to improve circularity in the e-waste industry. Bressanelli et al. [13] have called for studies focusing on companies and supply chains, to explore how to address the first three Rs (i.e., reduce, reuse, and remanufacture) in a 4R strategy and for a more systematic and holistic perspective on research related to both actors and products in e-waste. Actors in this context include manufacturers and PROs, whereas the product perspective includes the product lifecycle phases. The attention to the CE in the field of supply chain management (SCM) has recently increased, and there is a call for more research addressing the practical aspects of implementing circular supply chains (CSC) [17]. Moreover, despite increased attention to circular supply chain management (CSCM) and new technologies, there is still little circularity in supply chains in general, and in WEEE in particular [13]. More knowledge is required of how actors in these supply chains are dealing with information and the use of digital technologies in facilitating information flow to achieve circularity [18]. Based on a comprehensive literature review within WEEE or e-waste, Islam and Huda claimed that most studies in the closed-loop supply chain (CLSC) are based on generic frameworks, and called for empirical research based on real-world scenarios [19]. Furthermore, Aarikka-Stenroos et al. [20] recently claimed that research on CSCM is still in its infancy, calling for more empirical investigations. There is a particular need for more qualitative case studies to provide an in-depth understanding of the practical problems related to e-waste handling [19]. In turn, this could lead to theory development [21]. Within this stream of literature, there is a need for research on the flow of information among actors in the e-waste industry, and on how new technologies, such as the Internet of things (IoT) and information technology-based systems, could improve the information flow in e-waste handling [19, 22, 23]. This includes research on the use of information systems to improve circularity, both to understand circular material flows and to support increased circularity at the beginning of life (BoL, i.e., circular product design), middle of life (MoL, i.e., intensified and extended product use), and EoL (i.e., material reprocessing) phases [24]. Knowledge of how technology can contribute to collection and transfer of information in order to achieve greater circularity is needed, including different actors’ general motivation and specific motives for supplying relevant lifecycle information.

This study contributes by addressing research gaps in the study of how product lifecycle information flow is handled in e-waste treatment in Europe by investigating a real-world product lifecycle information flow. A multinational EEE manufacturer with eight subsidiaries and five PROs was studied to provide real-world examples of how product lifecycle information flow is currently handled in the European EEE industry. The study is guided by the following research question:

How are EEE manufacturers dealing with product lifecycle information flow to increase circularity in e-waste handling?

The remainder of the study is structured as follows. In the “Literature Overview and Background” section, we present the current state of research on CSCM, e-waste, and digital technologies. The “Methodology” section describes the research methodology, followed by the results in the “Results” section. The “Discussion” section contains the discussion, the “Conclusion” section contains the conclusion part before the limitations, and future research directions is presented in the “Limitations and Future Research Directions” section.

## Literature Overview and Background

### Circular Supply Chain Management (CSCM) and Information

The CE represents an alternative to the traditionally dominant linear take-waste-dispose production model [25, 26]. The CE literature is fragmented and spread across several established fields ranging from environmental economics to management science [17]. Different streams of literature have emerged addressing different aspects of circularity and offering various approaches to achieving it [20]. CSCM integrates the philosophy of the CE model into SCM [27]. CSCs represent a transformation from a linear to a circular flow of products [28]. Farooque et al. [27] provided a comprehensive definition of CSCM (p. 884):

Circular supply chain management is the integration of circular thinking into the management of the supply chain and its surrounding industrial and natural ecosystems. It systemically restores technical materials and regenerates biological materials toward a zero-waste vision through system-wide innovation in business models and supply chain functions from product/service design to end-of-life and waste management, involving all stakeholders in a product/service lifecycle including parts/product manufacturers, service providers, consumers, and users.

**Fig. 1:**
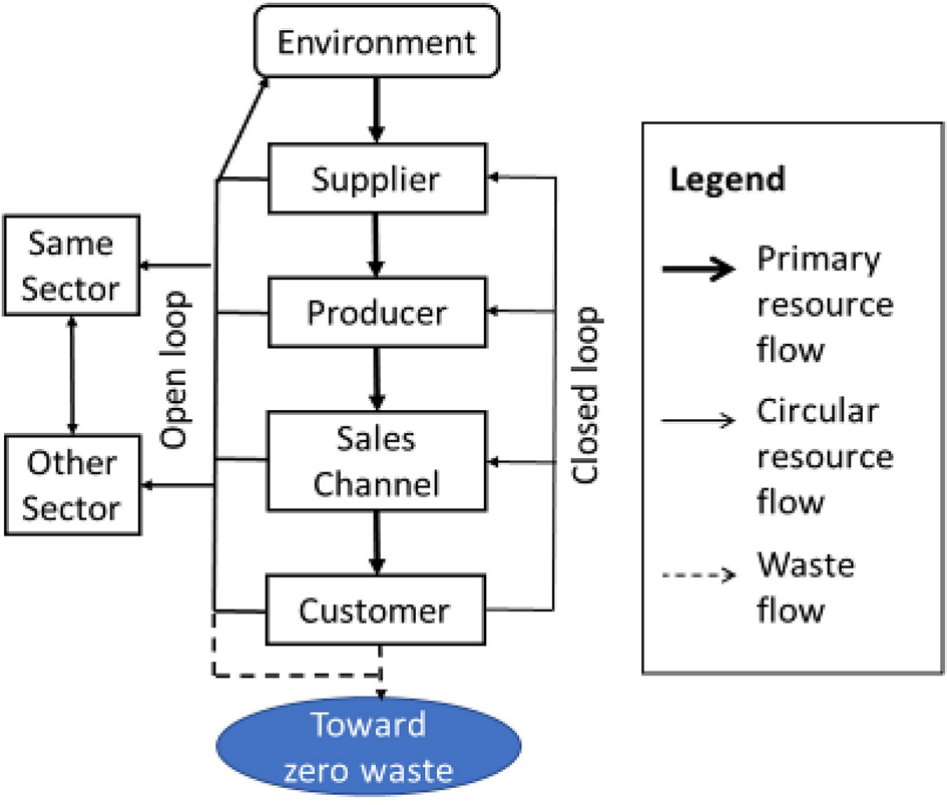
Circular supply chain [27] (p 885)

This definition emphasizes the regenerative dimension, which applies to circularity in manufacturing products and services. Notably, the circularity concept includes the time dimension to ensure the protection of future generations from economic, environmental, and social concerns [29]. Moreover, CSCs differ from CLSCs [30] because they emphasize the recovery of value from waste through collaborating with other organizations, either in the same sector or in other sectors and supply chains. Consequently, waste unsuitable for reuse in one manufacturer’s supply chain could be used in others. In an open-loop supply chain (OLSC), the actors bringing products to the market are not those that retrieve the products after EoL for reuse/recovery [31, 32]. Figure 1 illustrates the principle of a CSC.

Several research streams discuss various CSCM aspects. For example, Aarikka-Stenroos et al. [20] divide the literature on collaboration in implementing CSCM strategies into four streams: production and manufacturing research; supply chain, operations, and logistics management research; sustainability and CE research; and industrial business and B2B research. Farooque et al. [27] address the integration of CE into different supply chain functions as product/service design, procurement, production, logistics, consumption, and EoL and waste management. Within this category, they are mentioning appropriate treatment of EoL products (particularly WEEE) as a relevant issue in the transition toward CE. This study contributes to this literature, and in particular how product lifecycle information flow is handled in e-waste treatment in Europe.

Information flow and connectivity are crucial for the optimal performance of CSCs [33, 34], possibly affecting several of their key elements, such as strategy, structure, and flow [17]. Integrating the information flow is crucial in realizing the environmental and economic value of the discarded products [34]. Circular product lifecycle information originates from various actors in the product lifecycle, ranging from product development to EoL actors, and includes tacit knowledge, practical experience, paper manuals, software, pictures, and advanced specifications. However, despite the importance of information in CSCM, circular information flow essentially remains to be established. Reported constraints include lack of interaction with remanufacturers for information exchange and sharing due to confidentiality reasons, lack of knowledge-sharing platforms, and lack of data-transferring channels between actors [35]. Possible initiatives to address these constraints have been proposed, such as developing standardized data-sharing channels, establishing accessible knowledge-exchange platforms, increasing data exchange speed through tied collaboration between actors, and expanding data ownership in a system of shared values [35]. Hence, digital technologies may play an important role in achieving the information flow required for circularity, which we address in the “CSCM and Digital Technologies” section. Product lifecycle information and the associated information flows can be divided into the stages of a product lifecycle during which the information is established or used, for example, the product development, manufacturing, and logistics and maintenance phases [36]. This indicates that product lifecycle information is useful throughout the product lifecycle.

**Fig. 2:**
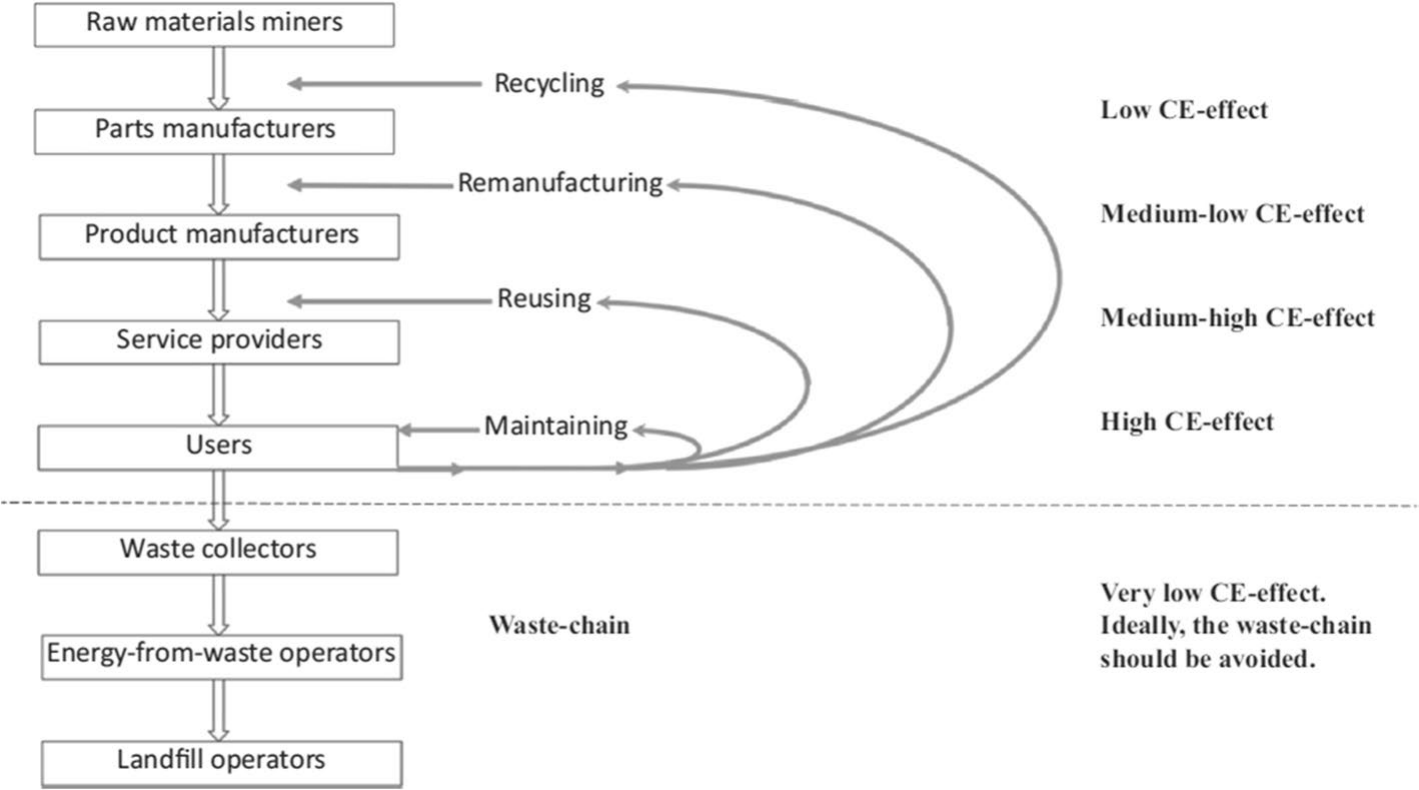
Schematic of CE as a restorative system for technical products, showing the M-3R cycles in the upper part and the waste chain in the lower part [37, 42]

Figure 2 illustrates the different CE strategies in a maintenance, reuse, remanufacture, and recycle (M-3R) model, including a waste chain [37] that categorizes the CE initiatives into levels of CE effects [38]. The figure shows that maintenance has the greatest impact on circularity, followed by reuse, remanufacturing, and recycling. The waste chain has a low impact on the CE. Assuming that the CE depends on information sharing, more information is required for a high CE effect. For supply chains, information flow is one of the three important flows; material and financial flows are the other two [39]. Although standards and protocols have been established for supply chains, this is not always the case for reverse supply chains [33, 34]. In this study, reverse SCM is defined as the activities required to retrieve a product from a user and either dispose of it or engage in reuse/recovery [40, 41]. This can be a part of a CSC, but this is not always the case. According to Farooque et al. [27], CSCM involves circular thinking in addition to some ambitions related to CE strategies, while reverse SCM does not always share these ambitions.

### CSCM and Digital Technologies

Digital technologies are vital for collecting and transferring the information required to establish CSCs. However, research on how emerging technologies can support the transition to CSCM is in its infancy [27, 43, 44]. Previous studies have connected the use of digital technologies to reduce, reuse, recycle, and restore (4R) approaches [38, 45]. The importance of information flow for more circular strategies, such as remanufacturing, has been well documented [38].

Based on a literature review, Bressanelli et al. [10] identified several digital technologies, such as IoT, big data analytics, cloud computing, 3D printing, blockchain, and augmented or virtual reality, as CE enablers. According to them, digital technologies can be designed to

- facilitate access to data that supports product management throughout its entire lifecycle, lifetime extension and optimization, provision of spare parts, enhanced understanding of user behavior, technology for service-based business models, decision support for the selection for the most suitable circular strategies at the end of use phases, and so forth. (p. 4)

A unique product identification ensures that product and information flow can be managed at the item level. Unique identification and labeling by manufacturers throughout the value chain enable implementation of cost-efficient circularity actions. Such identification can link a product to digital technologies, for example cloud technology, which is used to set up platforms on which information from all lifecycle stages can be shared [46]. Other examples are the application of radio frequency identification (RFID) technology to improve recycling operations and information for decision making [47] and blockchain technology and smart contracts in the development of CSCs [48]. Moreover, Wang and Wang [49] advocated the use of digital twins to provide lifecycle information in WEEE treatment. Together with Industry 4.0 enablers, such as smart monitors, wireless sensor systems, and other IoT devices, the digital twin “provides rich information to support the production and recovery operation throughout the product lifecycle” (p. 3895). Even if the identifier is static, the cloud system or digital twin may have updated information, allowing continuously updating of EoL treatment strategies. The global standards organization GS1 has developed standards for unique product identification based on both barcodes (i.e., the Global Trade Item Number [GTIN]) and RFID via the Electronic Product Code (EPC) [50]. These standards can link a product to the relevant product lifecycle information provided by relevant actors along the supply chain. Furthermore, Lindkvist and Sundin [51] see product–service systems/servitization as a solution for better lifecycle information in the use/MoL phase of a product. If the manufacturer sells the service/purpose of the product, it has control of the product in all lifecycle phases and has incentives to apply different DfX methodologies, such as design for service and design for remanufacturing.

The above studies are mainly characterized by exploration of the opportunities enabled by emergent digital technologies. Despite considerable interest in emerging digital technologies, few studies demonstrate how these technologies have been implemented to achieve greater circularity in supply chains. Consequently, it is suggested that the role of digitalization in enabling supply chain circularity should be investigated [10]. Moreover, additional knowledge of how technology can contribute to the collection and transfer of the required information in order to achieve greater circularity is needed. For this purpose, the information flow between actors must be explored. To address these issues, this study investigates how product lifecycle information flow is used in the forward supply chain of EEE and the RSC of e-waste.

### EEE Supply Chain and the WEEE Directive

E-waste has attracted significant attention from the scientific community and has been examined in several literature reviews. Although most of the relevant publications are categorized as environmental research, e-waste has also been studied from other perspectives [52]. Much of this research has examined metal recycling and recovery [14], the least preferred alternative in a CE model [42]. For example, Pérez-Belis et al. [53] divided e-waste studies into different categories, including waste management, waste generation, waste characterization, social aspects of e-waste, reuse of EEE, and economic aspects. In Europe, the problem of e-waste is regulated by the WEEE Directive [6], which has increased recovery and recycling targets over the past 20 years. Since 2015, the Directive has also included preparation for reuse among its targets, indicating an increased focus on circularity in the EEE industry [15].

In addition, e-waste has been studied as a logistic phenomenon. Islam and Huda [19] performed a comprehensive literature review of e-waste related to reverse logistics/CLSC issues. They identified 157 studies related to these issues from 1999 to 2017. However, even with the increased focus on circularity in dealing with e-waste, the EoL phase of EEE is still mainly regarded as waste [15, 54, 55]. Raudaskoski et al. [55] were the first to coin the term “circular electric and electronic equipment” (CEEE), shifting the focus from waste to circularity. The transition to a more circular product focus assumes that the effort commences in the design phase of a product. The European Eco-Design Directive [56] regulates the energy consumption during products’ use phase. Design for disassembly, dismantling, recycling, and recovery (DfX) has been proposed for both the EEE industry [57] and more generally [58]. As one respondent in a recent case study [55] stated, “the point of manufacturing and design is where it all starts. Whatever they do at that point will determine how the product will be used, re-used, and re/upcycled” (p. 24).

In a circular approach, e-waste may be regarded as material of actual or potential value. While the WEEE Directive emphasizes recycling and recovery, the European waste hierarchy [59] divides waste treatment into five categories: disposal, recovery, recycling, preparation for reuse, and, most importantly, prevention (non-waste, in this context). Potting et al. [38] expanded this list to 10 CE approaches: recover, recycle, repurpose, remanufacture, refurbish, repair, reuse, reduce, rethink, and refuse. These 10 approaches are further grouped into three overarching levels: smarter products or production, extended product lifetime, and useful material applications. The European WEEE Directive requires all EEE manufacturers to label their products with a WEEE symbol. This symbol indicates that the equipment must be collected and sent to specific facilities for recovery and recycling. The Directive also requires all EEE manufacturers to provide information about preparation for the reuse, maintenance, upgrading, refurbishment, and recycling of their products. The Directive states that [6]

- to facilitate the preparation for re-use and the correct and environmentally sound treatment of WEEE, including maintenance, upgrade, refurbishment, and recycling, Member States shall take the necessary measures to ensure that producers provide information free of charge about preparation for re-use and treatment in respect of each type of new EEE placed for the first time on the Union market within one year after the equipment is placed on the market. (p. 12)

Each Member State must determine how to implement this requirement [6]. The WEEE Directive allows manufacturers to delegate the operational EoL treatment to a third party, a PRO [8, 9].

### Summary

**Fig. 3:**
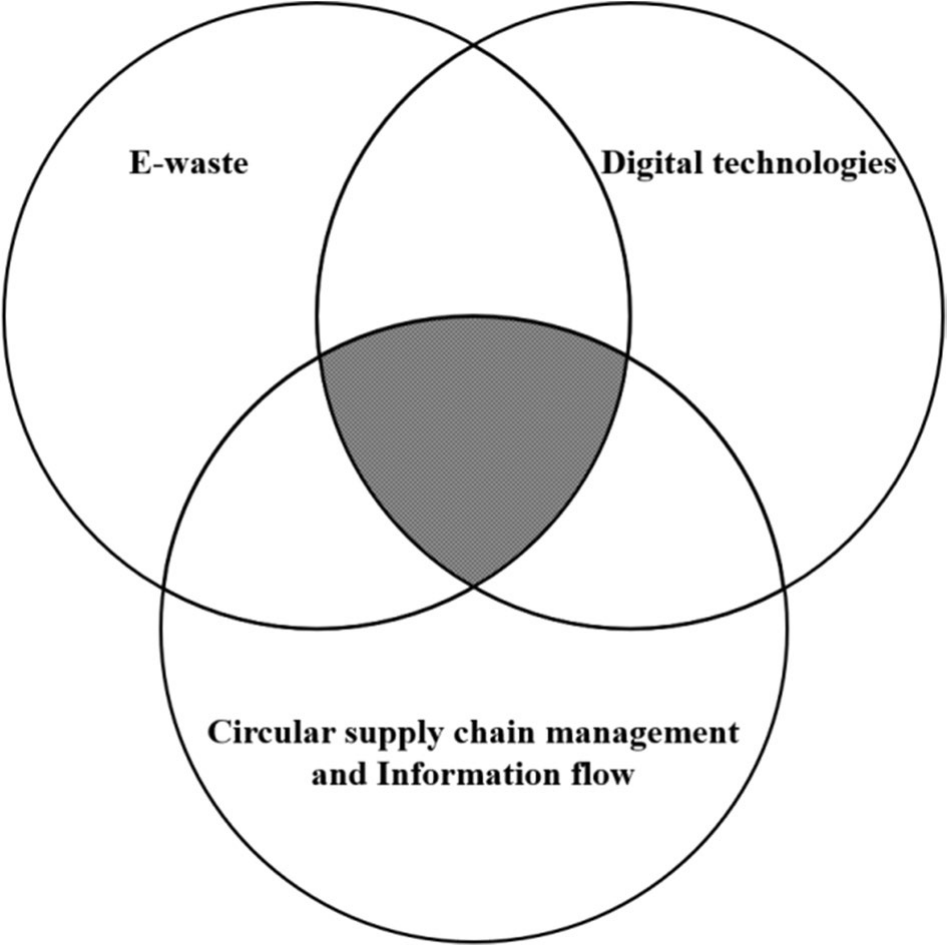
Theoretical foundation

This section has briefly presented the literature related to CSCM, and its relationship to information, digital technology, and WEEE/e-waste. Figure 3 illustrates that the focus of this study is on the intersection of the fields e-waste handling, CSCM and information flow, and usage of digital technologies to improve circularity. While the importance of information flow in regular SCM is well documented [36], research on this flow in CSCM is limited. Within SCM, usage of digital technologies to support this flow has been known for decades (e.g. [60].), within CSCM this knowledge is of newer date [10, 18]. E-waste handling in Europe has been regulated for more than two decades [6, 61], still the focus are on waste, not circularity [15].

## Methodology

The research question may call for several lines of enquiry concerning the actors’ general motivation and specific motives for supplying relevant lifecycle information, how they adopt digital technologies to manage such flows, and how they deal with this information. This study applies an open and broad approach to addressing these issues. The study is explorative in nature, as the research question is not well understood, and the research aims at providing insights into and an understanding of the research problem [62]. Hence, we conducted an inductive single-case study [63] exploring the product lifecycle information flow in EEE and e-waste handling, covering all these aspects. The selection of case is based on Flyvbjerg’s “information-oriented selection” [64], where the aim is to maximize the utility of the information from the case. Selecting critical cases permits logical deduction that produces new knowledge [64].

### The Case Company, Subsidiaries, and PROs

The case company is a multinational EEE manufacturer with manufacturing units and logistics hubs located globally. The company’s annual turnover is approximately USD 400 million, and it has approximately 2400 employees. On one hand, EEE constitutes international products typically produced in one country and exported to others. On the other hand, the export of e-waste is regulated and often prohibited. The case company was selected since it has several roles in EEE and e-waste handling and has a central role in providing and sharing information throughout the supply chain. The company operates as an EEE manufacturer, but also an importer and exporter of EEE. Some business processeus in the different units of the case company are common, such as product development and product certifications, while other processes are country specific, such as sales reporting and collecting environmental fees to cover the e-waste handling. The size of the company and the fact that it is present in many countries were important characteristics allowing us to obtain multiple results for comparison. Since the study was framed within the European WEEE legislation, we focused on the company’s activities in eight European countries: Belgium, Denmark, Estonia, Germany, Ireland, Norway, the Netherlands, and the UK.

**Fig. 4:**
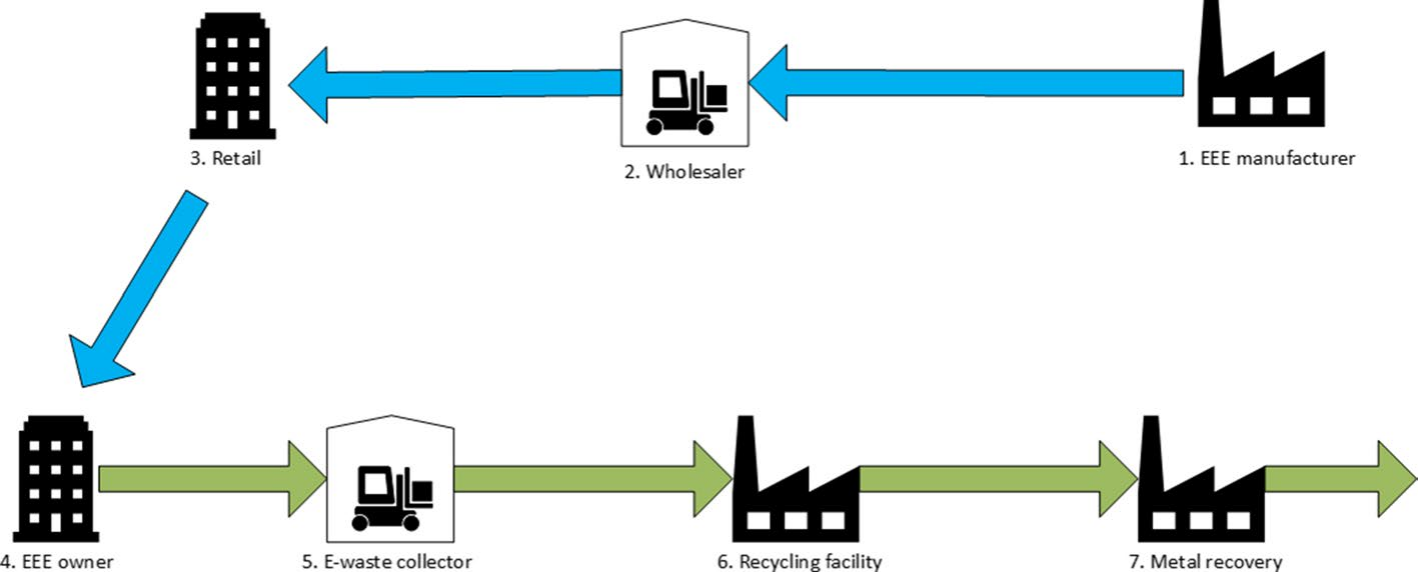
An example of a forward supply chain of EEE (blue) and the reverse supply chain of e-waste (green)

The case company operates in the business-to-business market and has factories in Estonia, Norway, and the UK that serve all eight countries, so this EEE manufacturer is a national manufacturer and exporter of EEE in three countries and an importer of EEE in all eight countries. In each country, the case company has an agreement with a PRO responsible for the collection and EoL treatment of e-waste. We received no responses from either the PRO or case company representatives in some countries, and the PROs in some other countries did not want to participate in the study. This has affected the geographical distribution of the study. The case company operates in an OLSC, meaning that there are different actors in the forward and reverse supply chains. However, in the financial flow for e-waste treatment, the chain is closed, i.e., a CLSC. The case company collects an environmental fee when selling EEE to finance its PROs. Figure 4 illustrates parts of typical forward and reverse supply chains in the EEE industry. Goods are shipped from the EEE manufacturer via a wholesaler and a retailer to the customer, represented as the EEE owner. The WEEE collector, recycling facility, and metal recovery represent the reverse supply chain of e-waste.

The EEE manufacturer is a significant actor within the EEE industry, acting as a manufacturer, importer, and exporter of EEE. Still, it is only handling some of the EEE categories. The PROs are all important organizations within e-waste handling in their respective countries, accounting for a significant amount of e-waste handling. Consequently, the case in this study can be seen as a critical case [64] in analyzing product lifecycle information flow in e-waste handling. This again can be used for analytical generalization [63].

### Data Collection

The data collection was based on semi-structured interviews with employees of the case company, including the e-manufacturing subsidiaries as well as employees at the associated PROs. The interviews were conducted during several periods in 2020. Due to the COVID-19 pandemic restrictions, all interviews were conducted by telephone or videoconferencing after March 2020. The interviews were recorded, transcribed, and sent to the case company for approval. All the case company’s subsidiaries and the PROs are unique legal entities. In total, 17 respondents were interviewed, representing 12 legal entities in eight European countries. At least one respondent in the case company in each country is responsible for implementing and monitoring adherence to the WEEE regulations in their country. A list of respondents, including their geographical distribution and roles, is presented in Table 1. The number of interviews refers to the initial phone or physical interviews. In addition, there were several follow-up questions by e-mail or phone.

**Table 1:** The interviews

Interview#	Country	Company	Participants	Number of interviews
1	Norway	EEE manufacturer	Accounting manager Responsible for WEEE reporting Quality manager Product approval manager Product development manager	4
2	Norway	PRO	Marketing and communication manager	1
3	Denmark	EEE manufacturer	Marketing manager	2
4	Germany	EEE manufacturer	Logistics manager	1
5	The UK	EEE manufacturer	Product manager	1
6	The UK	PRO	External affairs manager	1
7	Ireland	EEE manufacturer	Financial controller	1
8	Ireland	PRO	Compliance and membership manager	1
9	The Netherlands	EEE manufacturer	Financial controller	1
10	Estonia	EEE manufacturer	Financial manager	1
11	Estonia	PRO	General manager Management assistant	2
12	Belgium	PRO	Spokesperson	1

All interviewees from the case company were assured that no individual or company names would be mentioned in the study. Since EEE manufacturers and PROs have different roles in the forward and reverse supply chains, the opening questions/topics differed among the respondents. The interview topics are presented in Table 2. The questions were related to product lifecycle information and product lifecycle information flow. Initially, the interviewees were informed that the focus of this study was on improved circularity in EEE supply chains. We derived meaning from the interviews through content analysis, which enabled us to draw inferences by identifying certain characteristics in the data [65]. This open interview approach was necessary in order to obtain different perspectives on the research question. The interview results were analyzed in light of data gathered from business documents, which allowed for triangulation [65]. Business documents were mainly downloaded from the EEE manufacturers and PROs’ websites, but some were also obtained directly from the participants. The most important documents were product and marketing materials, such as certificates, manuals, declarations, and formulas for reporting EEE sales and e-waste. Results were coded to identify similarities and differences in interviews and business documents. Access to the resources required for the successful completion of this research was directly granted because one of the authors had, until recently, worked at the case company.

**Table 2:** Interview topics

Issue #	Respondents	Opening question/topic
1	EEE manufacturer	In addition to the WEEE icon, how are you providing or demanding extra WEEE product information following the goods (labeling)? Please give examples
2	EEE manufacturer	Are you providing additional WEEE information about your products to customers or other downstream actors? If yes, please give examples
3	EEE manufacturer	How do you access WEEE product information from other actors upstream in the supply chain (i.e., suppliers)? Please give examples
4	PROs	How are the recycling plants or collection points using information from the producers/importers to identify products and the content of products?
5	PROs	How would marking products with unique identifiers (e.g., a QR code) make it possible to identify products and the content of products and improve the recovery, recycling, or reuse process?

## Results

In this section, the results of the case study are presented. We present the results of the interviews, first, with participants from the case company and its subsidiaries and, second, with those from the PROs. The topics follow the order shown in Table 2.

### EEE Manufacturer

#### Product Lifecycle Information Following the EEE

All the respondents were aware of the WEEE symbol on the product label. The label also includes a product number, production date, and approval icons. One interviewee stated that the label had to specify the manufacturer, which consumers could contact for further information. Another respondent remarked that implementing the WEEE Directive is a national requirement, which complicates providing extra information since the EEE manufacturer is international. This respondent also mentioned that it would be difficult to stock products since they had to be marked or labeled differently for different countries: “A common international WEEE regime with the same rules would be wonderful”, he stated (all translations into English by the authors). Currently, there are no differences in the products, labels, or packaging depending on where the products are stocked or sold. This contrasts with the European WEEE regime, which is implemented differently in each European country: as one respondent stated, “The factories are handling this—no special terms for our country”. In addition to the WEEE symbol, some respondents referred to the installation and maintenance manual, presented in the next subsection.

#### Product Lifecycle Information Flow Downstream in the Supply Chain

Several respondents referred to the WEEE information in the installation and maintenance manual. This manual is available inside the packaging of a new product and on the EEE manufacturer’s website. The WEEE statement in the manual is general, declaring that the product is subject to the Directive and that it should be treated according to the Directive at EoL. The statement is repeated in eight languages. The English version of a representative WEEE statement is as follows: “Our products are subject to the Directive 2012/19/EU (Waste Electrical and Electronic Equipment—WEEE) and should at the end of their lifespan always be collected separately and brought to the appropriate collection point in your community or region”.

Several respondents referred to their websites as sources of product lifecycle information. One respondent mentioned the products’ datasheets. Each product has a datasheet that describes the product, including electrical data and dimensions, and the weight and materials used, which are relevant to recovery and recycling strategies. The case company provides datasheets for all its products, and for products no longer produced or sold. The datasheets for phased-out products were not easy to access on the company’s website, but searching for the products by unique item identification (e.g., item number) gave access to these products’ datasheets. However, as the case company had completed several acquisitions, which increased its product portfolio, documentation associated with expired products from the acquired companies was often unavailable. Although the EEE manufacturer provided a significant amount of data for the products, both current and expired, none of it had specific WEEE information on EoL treatment except in the installation and maintenance manuals. The main purpose of the information provided on the EEE manufacturer’s website was to support new sales.

One observation concerning the information flow was the unclear responsibilities of the case company regarding the provision of e-waste information. The manufacturing division placed the responsibility on the country subsidiaries selling the EEE equipment, while the country subsidiaries selling the equipment referred to the manufacturing units. As one representative of product development/approval stated, “Return companies must have access to information about how the various EEE equipment is to be disposed of. I am not sure how this is done. This is the responsibility of the sales units”.

#### Product Lifecycle Information Flow Upstream in the Supply Chain

Four respondents referred to their suppliers’ websites for the product lifecycle information flow upstream in the supply chain, where product information regarding parts and products from other suppliers used in the case company is available. However, none of the respondents could identify specific WEEE information from the suppliers, only general information also expected for products outside the WEEE Directive. One respondent stated that they only used European suppliers, indicating that this practice secured the information required by the WEEE Directive. He stated that all European EEE suppliers had to comply with the Directive, which ensured that the case company was also compliant.

### Producer Responsibility Organizations (PROs)

The PROs have a different role, specializing in e-waste handling with one main mission, namely, to ensure that the industry complies with the actual rules and regulations related to e-waste handling. The respondents from the PROs had a deeper understanding and knowledge of the WEEE Directive since the main purpose of PROs is to process e-waste/WEEE.

#### Product Lifecycle Information Flow from EEE Manufacturers to PROs

We had expected that there would be a flow of product lifecycle information about recovery, recycling, and reuse conveyed by, for example, drawings, assembly and disassembly instructions, bills of materials, 3D models, repair manuals, and quality measurements. However, none of the interviewees confirmed that this information flow was regularly used. Three respondents indicated that the information flow was available in special cases, and one specifically stated that the recycling plants had their own databases regarding EEE and the contents of different EEE. Another respondent explained that handling this information could hamper the required performance of the recycling and recovery processes at the collection and recycling plants. All the respondents mentioned that the speed of the recycling and recovery processes is important, so it is important to avoid any delay. One respondent stated that, for example, in a startup situation, if there was an increase in a specific type of e-waste, return factories would collect and use product lifecycle information from EEE manufacturers to improve their competence. For example, the electricity providers in a country replaced manual electricity meters with automatic reporting meters at the national level to simplify the measurement of electric energy consumption. This change resulted in an increase in the recycling of the old meters at the recycling facilities. The recycling facilities contacted the meter manufacturers to determine the correct recovery and recycling approach for this equipment.

Two of the respondents indicated that because the WEEE Directive is a waste directive, the PROs focused on the lowest CE effects, i.e., recycling and recovery. Higher CE initiatives, such as remanufacturing, reusing, and maintenance, are being implemented, but this is outside the responsibility of the PROs since they are only involved when EEE becomes e-waste. This indicates that if the PROs become more involved in these CE initiatives, they would possibly request and use more product lifecycle information.

#### Unique Product Identification for Improved Product Lifecycle Information Flow

The noted performance focus on the recycling and recovery processes also affected the answers related to unique product information. Two respondents stated that the speed and performance of the recycling process were general challenges, and that anything that might affect this is regarded as negative. Another respondent claimed that the use of QR codes had to be considered part of a larger digitalization project in the sector, not only for the products. Two respondents were more positive and said that unique product identification could identify the waste stream. Currently, there is no link between how much EEE a manufacturer releases into the market and how much e-waste is generated by the same manufacturer. As one participant from a PRO stated, “We are the ones who can link manufacturers to recyclers”. Another interviewee from a PRO added that a product’s production date might be important information because it could indicate whether certain hazardous materials had been used, as regulations may prohibit the use of certain materials after a specific date. In addition, two interviewees from PROs explained that they had participated in research projects related to digital technologies, one related to IoT technology and another to the use of artificial intelligence for product recognition in the supply chain.

### Major Findings

The interview results can be summarized as four major findings. First, the EEE manufacturer provides a significant amount of product lifecycle information that is available to customers, suppliers, and other actors in the supply chain. The main purpose of this information is to support equipment sales, and the main platform for this communication is the EEE manufacturer’s website. However, some of this product lifecycle information is relevant to the EoL phase of products. Second, specific product lifecycle information regarding EoL treatment is limited, even close to nonexistent. The provided information is general and can also be provided by manufacturers not subject to the WEEE legislation. Third, the available product lifecycle information is not used by or is of limited usefulness to the PROs in the reverse supply chain. This is due to the speed and performance focus of the recycling and recovery processes. Last, the PROs do not perceive a need for unique product identification of EEE to improve product lifecycle information flow. Their argument recalls the finding above, which is that the focus is on speed and performance in the recycling and recovery processes. These results are summarized in Table 3.

**Table 3:** Summary of findings

Respondents	Topic	Finding
EEE manufacturer	General product lifecycle information upstream and downstream in the supply chain	A significant amount of general product lifecycle information is available via the different actors’ websites. This information is provided for purposes other than EoL treatment
EEE manufacturer	Specific product lifecycle information regarding the EoL treatment of e-waste	Very limited specific EoL product lifecycle information is available
PROs	Use of information from the producers/importers to identify products and their contents	Limited usage due to the need for high performance in the collection, recovery, and recycling processes
PROs	Unique product identification to improve recovery, recycling, and reuse processes	Interest is limited due to the need for high performance in the collection, recovery, and recycling processes

## Discussion

In the literature addressing circularity and CSCM, product lifecycle information has been identified as important for realizing higher circularity strategies, such as remanufacturing, refurbishment, repair, and reuse, either directly or by implementing digital technologies to improve the information flow [35, 49, 51, 66, 67]. The European WEEE Directive states that producers must provide information concerning reuse, and mentions the three CE strategies, namely, reuse, recycling, and recovery. This means that all manufacturers affected by the European WEEE legislation must report the number of new EEE products released into the market at the beginning of their lifecycles. At the end of the product lifecycles, PROs must report the collection rates of e-waste. The BoL and EoL reporting regimes differ between countries regarding, for example, the overall figures, detailed information about product types, and product contents [16]. Briefly, the current information provided by BoL and EoL reporting provides only a rudimentary overview of e-product handling, leaving governance agencies, manufacturers, and society at large in a void.

In this study, we investigated how actors in an EEE network comprising an international manufacturer with several subsidiaries and associated PROs deal with the product lifecycle information flow in order to improve circularity in WEEE supply chains. Twelve actors in eight European countries were interviewed about this topic. We found that the case company, an EEE manufacturer, provided a significant amount of product lifecycle information upstream and downstream in the supply chain, but at a rather general level. We found that more specific product lifecycle information useful for EoL treatment was limited, even close to nonexistent. None of the available product lifecycle information was provided specifically to support preparation for reuse, maintenance, upgrading, refurbishment, remanufacturing, or recycling.

An important finding of this study is that the PROs handling the e-waste make only limited use of the information provided by the manufacturers. They claim that using this information could hamper their performance in the collection, recycling, and recovery processes. Moreover, unique product identification linking the EoL treatment to the manufacturer is not regarded as useful by the PROs, for the same reasons associated with performance and speed. In the literature, digital technologies are considered important enablers of the CE transition [10, 68]. Digitalization may create opportunities to track EEE products in both the forward and reverse supply chains [49]. An example of such technology is unique product identification for tracking, using either sensors/IoT [69] or product labeling [70]. The studied PROs, however, do not regard this as a feasible solution for improved recovery or recycling processes as, in their view, it is possible to implement recovery and recycle strategies with a limited flow of product lifecycle information.

At the system or regulatory level, one reason for the lack of provision and use of product lifecycle information is that the main focus of e-waste handling, according to the European WEEE legislation, is actually on recovery and recycling [15, 16]. Another reason is the organization of e-waste handling in Europe, with the outsourcing of EoL treatment to PROs, which means that EEE manufacturers are decoupled from the EoL treatment of e-waste. These OLSCs in the EEE/e-waste industry have challenges other than CLSCs, challenges related to product lifecycle information flow, since the manufacturers are disconnected from the EoL treatment. EoL initiatives by manufacturers may therefore not filter through to the PROs.

Another issue is delays in information transfer from EEE manufacturers to e-waste collectors, recyclers, and remanufacturers, delays that may differ from product to product depending on the product lifespan. A washing machine used in Germany has an average lifespan of 12.5 years [71], while a smartphone may have an average lifespan of under two years [72]. Hence, product lifecycle information about the EEE may, depending on the product’s lifespan, have been produced before the current regulation was implemented.

## Conclusion

Transforming e-waste handling from mere waste handling to circularity calls for well-functioning product lifecycle information flowing upstream and downstream in supply chains [33, 73]. These flows should be valid in all product stages, from BoL via the use phase/MoL to EoL [74]. However, sensing, capturing, managing, and utilizing this information are challenging tasks [75, 76]. Digital technologies have been emphasized as enablers of the transition to CE [10], but we lack empirical studies of how sharing product lifecycle information and using information technology in CSCs can improve circularity [10, 22]. This study has addressed this gap by carrying out a qualitative case study of a multinational EEE manufacturer. We interviewed employees in the case company, its subsidiaries, and associated PROs to explore how the actors in this supply chain are handling product lifecycle information flow in order to improve circularity.

The main finding of this study is that there is a mismatch between the optimistic views in the literature of the role of digital technologies in improving circularity [10, 18], and our findings from the EEE/e-waste industry regarding how product lifecycle information is actually used. Even though product lifecycle information is used in specific situations, in this case when new waste streams arise, this information is not used on a regular basis. This indicates that digital technologies alone are insufficient to enhance circularity in this industry, and that structural and governmental changes are required before new technologies can be introduced to create CSCs in the EEE industry. To realize the opportunities afforded by digital technology, supply chain actors need incentives, and these incentives are currently missing in the reverse supply chain of e-waste. Furthermore, implementing higher CE standards will require initiatives from manufacturers, such as designing for remanufacture or reuse. The findings of this study contribute to the literature in the CE and CSCM field by highlighting the challenges of achieving increased circularity in existing value chains, despite an extensive regulative regime and availability of technology and information in the WEEE industry [18].

From a management perspective, it is important to know the limitations of EoL treatment according to the WEEE Directive. If EEE manufacturers increase their efforts to manufacture more circular products, these efforts must be utilized at EoL. Currently, product lifecycle information does not reach the PROs. EEE manufacturers’ initiatives to improve the circularity of products beyond recovery and recycling must currently be undertaken outside the European WEEE regime. Changes in supply chain structure, responsibilities, and incentives to facilitate product lifecycle information flow are also needed in order to achieve higher circularity. In the current e-waste regime, both PROs and EEE manufacturers are important actors in organizing these initiatives.

## Limitations and Future Research Directions

This case study covers one industry, eight countries, and 12 legal entities. The five included PROs represent a significant part of the reverse supply chain of e-waste in Europe. Although generalizing from a single-case study may be possible in certain scenarios [63, 64], the present study has limitations in this regard. This study focuses on an EEE supply chain and the associated reverse supply chain of e-waste. The findings are likely to also be valid in other OLSCs. However, this case study only examined e-waste in Europe and included only some EEE categories. Expanding the study to encompass other cases dealing with other EEE categories in other countries should provide more insight into this issue. Our focus was on e-waste handling according to the European WEEE Directive. There may be flows not covered by this Directive that would also be valuable to study, for example, solutions for secondhand EEE or the remanufacturing of EEE beyond the WEEE Directive.

Further studies should focus not only on the general potential of digital technologies but also on how forward and reverse supply chain actors could benefit from these technologies to facilitate product lifecycle information flow in supply chains for improved circularity. Further studies should also address the barriers to using product lifecycle information, and how the organization and governance of supply chains could enhance information sharing for CE strategies.

## Data Availability

This study has been registered with the Norwegian Centre for Research Data (NSD). Interview data, both recorded source material and the transcribed results, have been stored. According to the agreement with the NSD, data that can identify people will be anonymized or deleted by 30 September 2023 at the latest.
